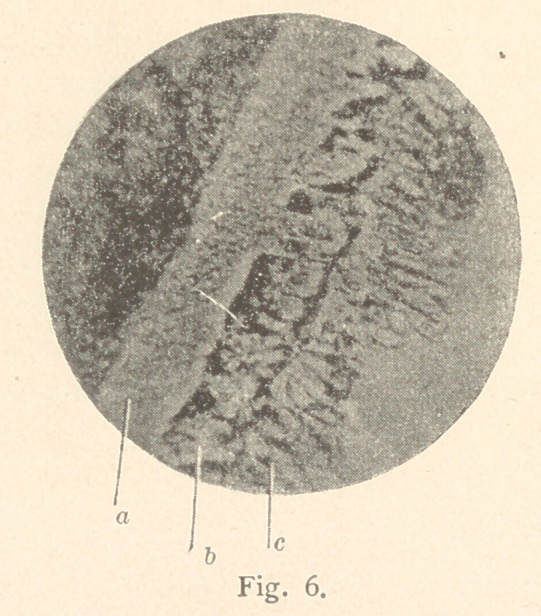# The Border-land of Calcification

**Published:** 1889-04

**Authors:** R. R. Andrews

**Affiliations:** Cambridge, Mass.


### International Dental Journal.

Vol. X. April, 1889. No. 4.

### Original Communications.[1]

### THE BORDER-LAND OF CALCIFICATION.[2]

BY R. R. ANDREWS, CAMBRIDGE, MASS.

In studying the formation of dentine with the higher powers of the microscope, in tissues which have been decalcified by the action of weak acids, there is found between what was the fully calcified tissue and the adjacent organic tissue from which it is formed, a peculiar layer, hyaline in its appearance, and which has been named calcoglobulin. In an investigation extending over several months, I have been much interested in studying its formation. The sections which I have prepared, that show this layer best, are cross sections of forming teeth, at a period when calcification is commencing, or perhaps better, on the edge of a calcifying dentine-germ at any stage before the tooth is wholly formed. The peculiar globular formations, next the formed layer of dentine, show best in tissue that has been in the decalcifying acid for two or three days only.

A brief description of the experiments of Prof. Harting and Mr. Rainie, showing the peculiar action of some of the salts of lime in albumen, may be of interest to us at this point; for they claim by these experiments to have found the explanation of the method of development of teeth, bone and shells. Mr. Rainie found that if carbonate of lime be slowly added to a thick solution of albumen, the resultant salt is in the form of globules laminated in structure like tiny

---

[1] The editor and publishers are not responsible for the views of authors of papers published in this department, nor for any claim to novelty, or otherwise, that may be made by them. No papers will be received for this department that have appeared in any other journal published in this country. The journal is issued promptly on the 15th of the month.

[2] Read at the Tenth Anniversary Meeting of the Odontological Society o Pennsylvania, Dec. 12th, 1888.

onions; the globules in contact become agglomerated into a single laminated mass, appearing as if the lamina in immediate apposition were blended with one another. The globular masses, at one time of mulberry-like form, lose the individuality of their constituent smaller globules, and become smoothed down into a single mass. Mr. Rainie suggests, as an explanation of the laminated structure, that the smaller masses have accumulated into concentric layers which have subsequently coalesced, and in the substitution of the globular for the crystalline form in the salt of lime when in contact with albumen, he claims to find a satisfactory explanation of the development of bone, teeth and shells. What he found was really the first stage in the process of the calcification of a tissue.

Prof. Harting has shown that the albumen left behind, after treatment of these globules with acid, is no longer ordinary albumen. It is profoundly modified, and has become exceedingly resistant to the action of acids, resembling chitine, the substance of which the hard skin of insects consists, rather than any other body. The small onion-shaped globular bodies, he has named calco-spherites, and the layer caused by the coalescing of these, calco-globulin, as it appears that the lime is held in some sort of chemical combination; for the last traces of lime are retained very obstinately when calcoglobulin is submitted to the action of acids, in the same manner as does that layer which is found everywhere on the border-land of calcification between the fully calcified and the formative tissue. In the course of my investigation I have found many sections showing the formation of these peculiar globular masses on the edge of forming dentine (see Plate, Figs. 3, 4, 5, and 6). One of my specimens shows the edge of dentine, which is to be covered by enamel, overlaid with small globules. (See Plate, Fig. 2.) These are calco-spherites. Those nearest the dentine have become a part of the matrix showing only a portion of their contour; others near them are spherical, of various sizes, and have a glistening appearance; some are made up apparently of a number of smaller ones. At a point a little above, in the same specimen, this time on the edge of the forming enamel, are seen elongated masses of this substance, made up of many small globules, or calco-spherites, which are losing their identity. This section is from a human fœtus in the sixth month. Among many cross sections that I have prepared from the tooth of a calf, at birth, there are some which show these globular formations very beautifully. If we examine another, using a low power, $\frac{1}{2}$ inch, we shall see the band of forming dentine to be about as wide as the layer of odontoblasts just within. The section has been stained with alum carmine, but has taken the stain faintly. Next the dentine, towards the pulp, and apparently among the odontoblasts, are seen, even with this low power, irregular glistening globular masses. At a point just below where these are seen, the pulp tissue and the odontoblasts have been pulled away from the layer of dentine, with no appearance of globular masses clinging to it. The edge has a glistening appearance, something like the globules mentioned above; under a high power, 1-12. Im. obj., this glistening edge shows rounded contours, as if there had been globules which had become part of the already formed band of dentine. In the substance of some of the odontoblasts, and even in the tissue of the pulp near them, are seem small glistening globules, calcospherites.

In another section the narrow forming band of dentine is seen to be made up mostly of globular masses. See Plate, Fig. 3. These are especially bright toward and among the odontoblasts. Nearest the pulp they have the glistening appearance which is seen in fat cells. In still another section these globules are in line and have nearly formed what is to be a new layer of the dentine matrix. They have taken the stain nearly, if not quite as well, as the dentine already formed, and commence to look very much like it. In places against the formed dentine some of them have, where they were against it, become a part of it, merging into it, without any line of separation whatever. (See Plate, Figs. 4 and 6.) Smaller globules appear to be imprisoned between them, nearest the dentine, and these have a marked granular appearance. The forming layer is, at this very early stage of the formation of the dentine, about as wide as the layer of dentine formed, and is also about as wide as the layer of formative cells—the odontoblasts—sometimes, though wrongly, called the membrana eboris. At a later stage, when the calcified layer of dentine is thicker, the layer of calco-globulin is much narrower; and while I have never been fortunate enough to observe it forming in this manner, yet indications of globules and globular masses are never difficult to find within the layer of calco-globulin.

I have been somewhat interested in this connection by a paper read by F. J. Bennett before the Odontological Society of Great Britain, "On Certain Points Connected with the Structure of Dentine." Having become interested in some of the experiments of Dr. Miller, Ord., in which it was stated that pieces of ivory became eroded if immersed in a solution of subcarbonate of potash in glycerine, Mr. Bennett experimented with tooth structures under similar conditions. First, freshly extracted teeth were ground sufficiently thin to allow microscopic examination, and were then immersed in glycerine, or one of the carbonate or subcarbonate solutions (as above), and after various periods—one to six months—examined in glycerine. The dentine was found to have become transparent at the margin of the pulp chamber of longitudinal sections; the adjacent dentine was seen, under a low power, to be fringed and laminated. Under $\frac{1}{8}$ inch obj. this appearance was seen to be due to the dentinal tubes having lost their intertubular tissue. The course of the tubes appeared further to be interrupted at regular intervals by layers of what Mr. Bennett calls membranes having a direction parallel to the surface. These layers resemble the appearances seen in interglobular dentine; but circular apertures replaced solid globules, and oval spaces existed between the layers. Through these circular apertures dentinal tubes could be seen crossing from one layer to another, and completely freed of intertubular tissue. The tubes seemed to be measured off regularly into short lengths by the crossing of the layer

Later on he says, " Glycerine had clearly acted, but not destructively, since changes were brought about by it resembling normal developmental structures, it had acted selectively. Various explanations of the changes described were offered; among others, this: That the layers merely represented a part of the matrix itself, which resisted the action of the glycerine. The surface of the layers might present different stages of calcification, and thus offer a variable power of resisting the glycerine, the circular spaces representing portions which had been removed. This view, if correct, would accord with the theory of globular calcification in dentine. Interglobular dentine, if submitted to glycerine action, shows appearance of a membrane around the globules, and this fact supports the above given theory."

So much for Mr. Bennett's paper. It is somewhat difficult to arrive at exact conclusions in regard to this globular formation of the dentine. My investigation leads me to believe it is the first form that exists, previous to a calcified layer; that is, that small globules coalescing, form large ones, and these again coalescing, form the layer of calco-globulin which, by complete calcification, forms the dentine matrix or basis substance. While there are seen small glistening bodies, calco-spherites, in the pulp tissue near the odontoblasts, it is probable that the ones which form the larger globular masses have, for their source, the odontoblasts. In many places there is an appearance as though the odontoblasts were being enveloped in the larger globular masses that are forming the layer of calco-globulin, and which become, by calcification, the basis substance of the dentine. I am not, as yet, certain of this, however.

### DESCRIPTION OF PLATE

FOR

### DR. ANDREW'S ARTICLE

ON THE

### BORDER LAND OF CALCIFICATION.

---

FIG. 1. Layer of formed dentine, A. Calco-globulin, B. Odontoblastic layer and pulp tissue, C. Cross section calf tooth at birth, magnified 200 diameters.

FIG. 2. Outer edge of formed dentine, A. Small globular masses, calco-spherites, B. Pulp tissue, C. Tooth of human fœtus, 6th month, vertical section, magnified 1200 diameters.

FIG. 3. Forming band of dentine, A. The layer is seen to be made by the coalescence of the globular masses, B. Odontoblastic layer and pulp tissue, C. Cross section calf tooth at birth, magnified 1200 diameters.

FIG. 4. Band of formed dentine, A; with buds of calco globulin forming new layer, B. Odontoblastic layer and pulp tissue, C. cross section calf tooth at birth, magnified 1200 diameters.

FIG. 5. Band of formed dentine, A, with large masses of calco-globulin forming new layer, B. Odontoblastic layer and pulp tissue, C. Cross section calf tooth at birth, magnified 1200 diameters.

FIG. 6. Band of formed dentine, A; with buds of calco-globulin forming new layer, B. Odontoblastic layer and pulp tissue, C. Cross section calf tooth at birth, magnified 1200 diameters.

## Figures and Tables

**Fig. 1. f1:**
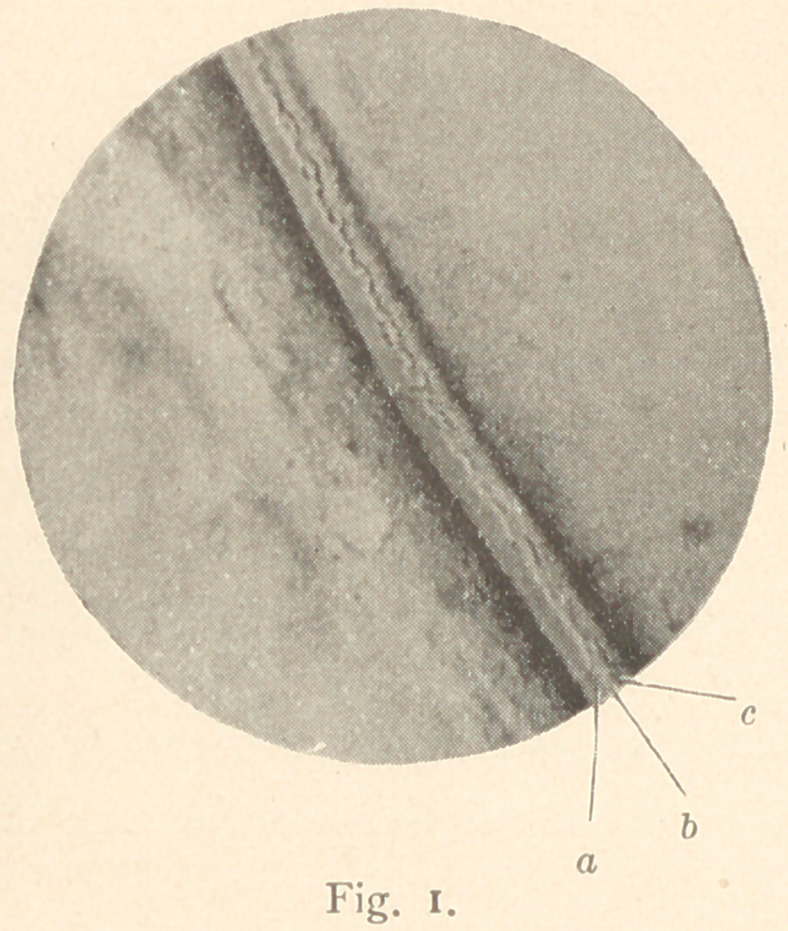


**Fig. 2. f2:**
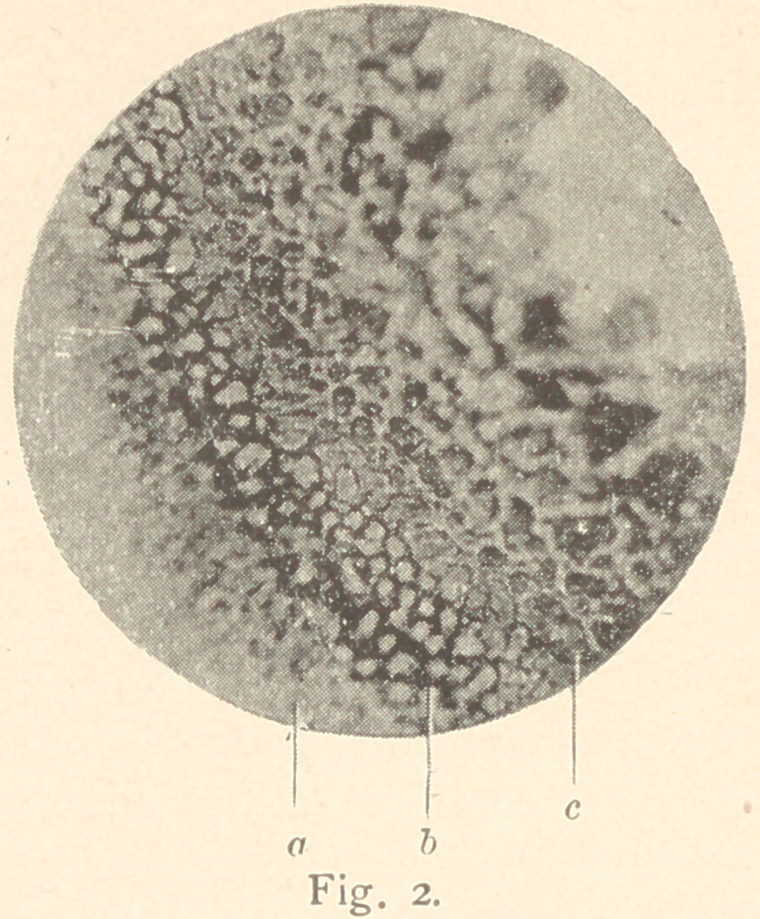


**Fig. 3. f3:**
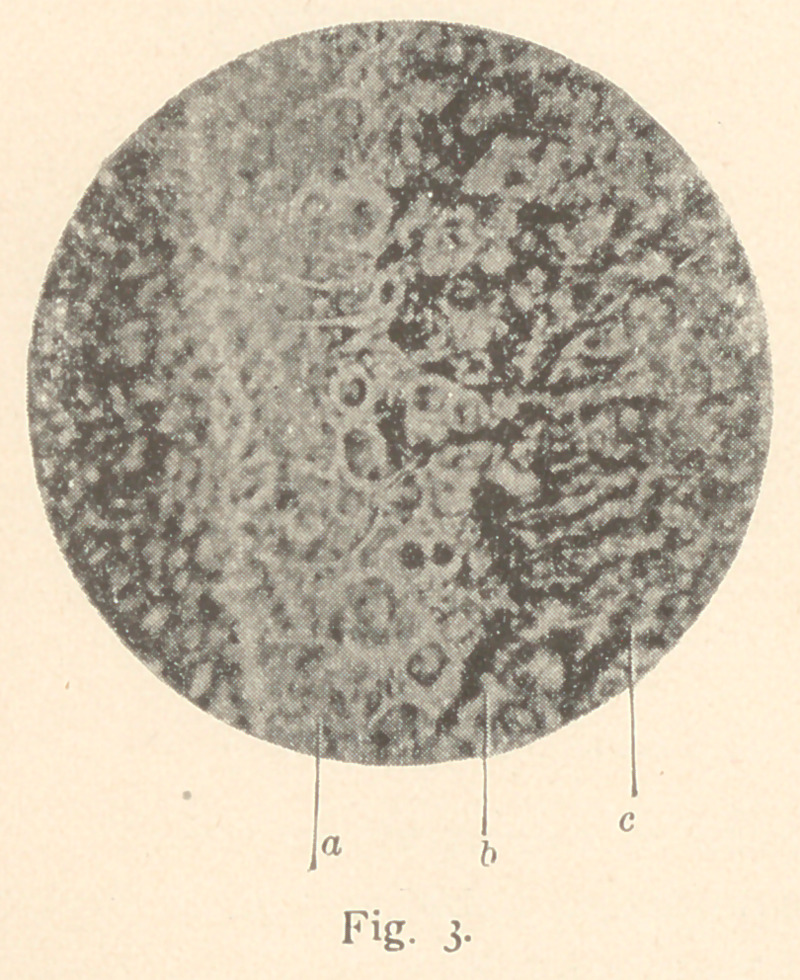


**Fig. 4. f4:**
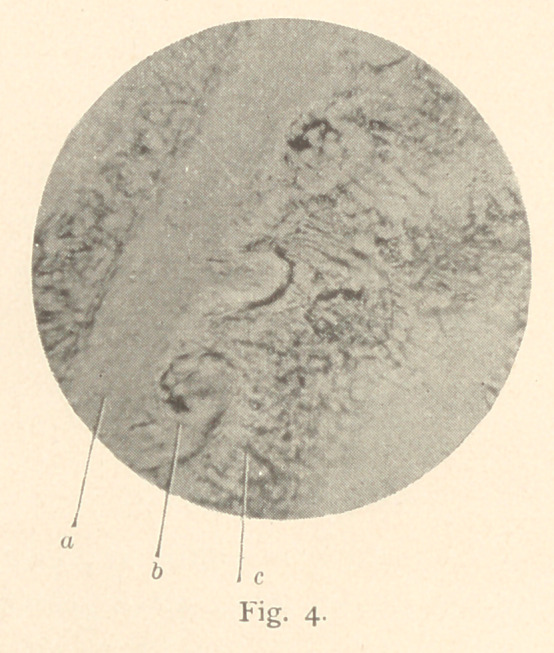


**Fig. 5. f5:**
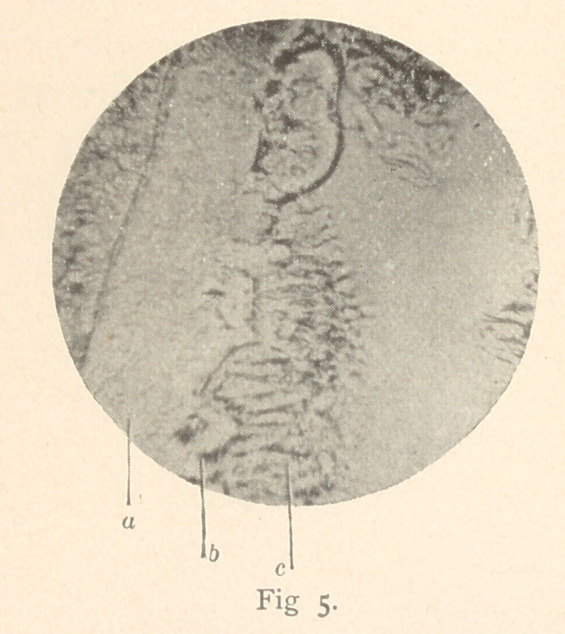


**Fig. 6. f6:**